# Genomic Diversity of the Major Histocompatibility Complex in Health and Disease

**DOI:** 10.3390/cells8101270

**Published:** 2019-10-17

**Authors:** Jerzy K. Kulski, Takashi Shiina, Johannes M. Dijkstra

**Affiliations:** 1 Faculty of Health and Medical Sciences, UWA Medical School, The University of Western Australia, Crawley, WA 6009, Australia; 2 Department of Molecular Life Sciences, Division of Basic Medical Science and Molecular Medicine, Tokai University School of Medicine, 143 Shimokasuya, Isehara, Kanagawa 259-1193, Japan; tshiina@is.icc.u-tokai.ac.jp; 3 Institute for Comprehensive Medical Science, Fujita Health University, Toyoake, Aichi 470-1192, Japan; dijkstra@fujita-hu.ac.jp

## 1. Introduction

The human Major Histocompatibility Complex (MHC) genes are part of the supra-locus on chromosome 6p21 known as the human leukocyte antigen (HLA) system. This genomic complex consists of more than 250 annotated genes and expressed pseudogenes usually partitioned into three distinct regions known as Classes I, II and III. Some of these MHC genes are located closely together in diverse haplotype blocks or clusters that are involved in encoding proteins for cellular and extracellular antigen presentation to circulating T cells, inflammatory and immune-responses, heat shock, complement cascade systems, cytokine signalling, and the regulation of various aspects of cellular development, differentiation, and apoptosis. In addition, there are hundreds of putative microRNA, long noncoding RNA (lncRNA) and antisense RNA non-protein coding loci within the HLA genomic region that may be expressed by different cell types and play important roles in the regulation of immune-response genes and in the aetiology of numerous diseases [1,2,3,4,5,6]. Since about 2010, the next generation sequencing revolution has been contributing slowly to a better understanding of human MHC gene diversity in worldwide populations, non-coding region variation of HLA loci, the effect of regulatory variation on HLA expression, diversity and polymorphisms in shaping lineage-specific expression, and the impact of HLA expression on disease susceptibility and transplantation outcomes [7]. There is considerable diversity of the MHC genomic region within and between different jawed vertebrate species and much of this diversity is found in the large structural and architectural differences in the genomic organisation of the MHC Class I, II and III genes [8,9,10,11]. The MHC of all jawed vertebrate species is characterised specifically by two primary classes of glycoproteins that bind peptides derived from intracellular or extracellular antigens to present to circulating T-cells and play an integral role in adaptive and innate immune systems [12]. Because of the MHC Class I and II gene sequences, duplications and functional diversity, the use of animal experimental models such as macaque, mice, quail, fish, etc., to evaluate the importance of the structure, diversity, expression and function of these genes in immunity, reproduction, mate choice, health, disease, transplantation and vaccination is invaluable [13,14,15].

This Special Issue on the “Genomic Diversity of the MHC in Health and Disease” consists of eighteen papers with one commentary [16], five reviews [17,18,19,20,21], eleven research articles [22,23,24,25,26,27,28,29,30,31,32] and one communication [33]. These papers cover a broad range of topics on the genomic diversity of the MHC regulatory system in various vertebrate species in health and disease including structure and function; MHC Class I, II and III genes; antigen presentation; innate and adaptive immunity; neurology; transplantation; haplotypes; alleles; infectious and autoimmune diseases; fecundity; conservation; lineage; and evolution. Although this Special Issue is largely limited to the MHC of mammals, birds and fish, with no expert paper provided on the MHC of monotremes/marsupials, reptiles or amphibians, taken together, these articles demonstrate the immense complexity and diversity of the MHC structure and function within and between different vertebrate species. 

## 2. MHC Genomics, Functions and Diseases from Humans to Fishes

Ten of the 18 papers in the Special Issue are human related, starting with a commentary by Dawkins and Lloyd who provided an overview of the history of the discovery of the association between HLA Class I, II and III gene alleles and certain human autoimmune diseases such as ankylosing spondylitis, systematic lupus erythematosus, myasthenia gravis, and type-1 diabetes from the perspective of conserved population (ancestral) haplotypes [16]. The authors were critical of the modern genome-wide association studies that are based solely on SNP typing and recommended that all MHC genomics and SNP typing results associated with phenotypes or disease be defined as haplotypes, preferably through segregation in extensive family studies for a better understanding of the mechanisms and concepts between HLA genetics, function and phenotypes. A similar sentiment about segregation analysis was extended recently to the study and sequencing of two MHC Class I loci in European barn owls in an investigation of allele segregation patterns in families, showing that family studies not only help to improve the accuracy of MHC genotyping and haplotyping, but also contribute to enhanced analyses in the context of MHC evolutionary ecology [34,35].

Shiina and Blancher provided an extensive review on the use of Old World monkeys in experimental medicine to study the role of MHC polymorphisms in allograft transplantation of organs and stem cells, immune response against infectious pathogens and to vaccines, and various biological systems including reproduction [17]. They compared and expanded on the essential differences and similarities between the human and monkey genomic organisation of the MHC following from their previous comprehensive review comparing the MHC genomics of humans, macaques and mice [36]. They also pointed out the difficulties of reconstructing the complex MHC haplotypes in Old World monkeys by whole genome sequencing using short reads because of the complexity and large number of MHC gene duplications in these animals.

O’Connor and co-authors reviewed the current concepts of avian MHC evolution in the era of next generation sequencing and genomics, focussing on the use of MHC Class I and II sequences to evaluate their associations with fitness, ecological effects, mating preferences, and parasite resistance [18]. Their review refers to the MHC genes of many bird species rather than focusing solely on the chicken MHC, which is an avian MHC model reference that is not wholly representative of most birds. The authors discussed the phylogeny of MHC structural evolution across the avian tree of life, highlighting the enormous diversity between MHC Class I and II gene copy numbers in over 200 species. They concluded that, despite the many inroads made in the last 20 years with the advent of high-throughput sequencing in understanding MHC structure, diversity and evolution, significant improvements still are needed in assembling complete MHC regions with long-read sequencing to establish robust genetic and physical maps in exemplar lineages of birds and to provide anchor points for MHC studies in diverse species. 

The MHC Class I and II antigen presentation systems probably emerged in the gnathostome (jawed vertebrates) because these two particular adaptive immune systems are absent in agnathans (jawless vertebrates such as the lamprey and hagfish) and invertebrates [37]. The cartilaginous sharks are elasmobranch fish and the earliest extant representatives of jawed vertebrates with a functional MHC antigen presentation system already established before the emergence of the teleost (modern bony fish) [9,10]. In this Special Issue, Yamaguchi and Dijkstra provided a critical review of classical MHC Class I and II functional analyses and disease resistance in teleost (modern bony fish) and a detailed account of MHC polymorphism and haplotype variation [19]. The authors were critical of many MHC-specific genotype-phenotype association reports in teleost fish, especially of those that claimed an association between MHC Class II haplotypes and mating preferences. Concerning disease-resistance association studies, they only considered whole genome quantitative trait loci (QTL) analyses that were based on statistical reliability. The authors concluded that the teleost classical MHC Class I allelic variations cannot be explained only by selection for different peptide binding properties, and they hypothesised that the extremely divergent alleles may have been selected to induce a more rigorous allograft rejection. In addition, in this Special Issue, Grimholt and co-authors communicated their discovery of a new nonclassical MHC Class I lineage that was found in Holostei (primitive bony fish) and as a new, sixth lineage in Teleostei (modern bony fish) [33].

While three reviews of the MHC structure and function focus mostly on the MHC classical and nonclassical Class I and II genes [17,18,19], one review [20] and a research article [22] in this Special Issue specifically describe some of the genes in the MHC Class III region that are associated with the innate immune system, complement activation, inflammation and regulation of immunity [1,2,3,4]. Zhou and co-authors reviewed a cluster of four genes NELF-E, SKIV2L, DXO and STK19 (the NSDK cluster) in the human MHC Class III region that are involved in RNA metabolism and surveillance during the transcriptional and translational processes of gene expression [20]. These four genes seem to engage in the surveillance of host RNA integrity, in the destruction and turnover of faulty or expired RNA molecules or RNA viruses, and in the fine-tuning of innate immunity. The NSDK cluster is located between the complement gene cluster that codes for constituents of complement C3 convertases (C2, factor B and C4) and the humoral effector functions for immune response. The authors regarded these four genes as highly under-rated because the genetic, biochemical and functional properties for the NSDK cluster in the MHC have remained relatively unknown to many immunologists. Some related gene sequences were found in *Drosophila*, *C. elegans* and zebrafish, but their important roles in human carcinogenesis, infectious and autoimmune diseases are only starting to emerge.

Plasil and co-workers provided a synopsis of the emerging genomic sequencing data for the tumour necrosis factor (TNF) gene and the lymphocyte antigen 6 (LY6G6) multicopy gene family in the MHC Class III region of camels [22]. The LY6 proteins that also are encoded by the MHC Class III region of humans and mice contain a cysteine-rich domain, and they are attached to the cell surface by a glycophosphatidylinositol (GPI) anchor, which is involved in signal transduction. In a comparative and phylogenetic analysis of these gene sequences, the authors found that the camel TNFA and LY6G6 genes mostly resemble those of pigs and/or cattle, as part of their continuing contribution to constructing and improving the genomic map of the entire MHC region of Old World camels.

The human MHC genomic Class I, II and III regions spanning ~4 Mbp from the telomeric myelin oligodendrocyte glycoprotein (MOG) gene to the centromeric collagen type XI alpha 2 chain (COLL11A2) gene also harbour numerous putative microRNA, lncRNA and antisense RNA non-protein coding loci that receive little or no investigative attention [5,6]. Kulski reviewed the origin and structure of the HCP5 gene located between the MICA and MICB genes of the MHC Class I region [21]. This lncRNA gene is a hybrid structure carrying the MHC Class I promoter sequences for the expression of a fossilised endogenous viral sequence ERV16, a repeat sequence that is widely distributed across the genomes of primates and some other mammals. Kulski also found that the HCP5 gene probably expresses the small protein PMSP that binds to the capsid protein of human papillomaviruses. Although the PMSP amino acid sequence appeared to be limited mainly to humans, its homologue was found recently in the baboon (Madrillus genomic sequencing project, UniprotKB: A0A2K5XZB9). Many recent studies have shown that HCP5 SNP sequences are strongly associated with various chronic and infectious diseases including HIV and that the HCP5 RNA interacts with genes inside and outside the MHC genomic region especially with microRNA in the regulation of different cancers. This review highlights the importance of gaining more information and a better understanding of the many noncoding RNA genes expressed by the MHC region that can affect health and disease in association with or independently of the MHC classical Class I and II genes.

## 3. MHC Classical and Nonclassical Class I and Class II Genomic Diversity (Haplotypes) and Peptide Presentation in Health and Disease

Five research papers are specifically on the topic of MHC antigen presentation and/or interactions with receptors of T cells or killer cells in health or disease [23,24,25,27,28]. One research paper focusses on haplotyping Class II genes using SNPs associated with disease [26], whereas another examines the importance of MHC Class I gene expression on spinal motoneuron survival and glial reaction following a spinal ventral root crush in wild type and beta2-microglobulin knockout mice [29].

The interaction between T-cell receptors (TCRs) and antigenic peptides presenting major histocompatibility complexes (pMHCs) is a crucial step in adaptive immune response. It triggers the generation of cell-mediated immunity to pathogens and other antigens. The response is driven by TCRs specifically recognising antigenic peptides bound to and presented by the MHC molecules of infected or transformed cells [12,13]. In this Special Issue, Karch and co-workers presented a molecular dynamics simulation study of bound and unbound TCR and pMHC proteins of the LC13-HLA-B*44:05-pEEYLQAFTY complex to monitor differences in relative orientations and movements of domains between bound and unbound states of TCR-pMHC [23]. They found decreased inter-domain movements in the simulations of bound states when compared to unbound states; and increased conformational flexibility was observed for the MHC alpha-2-helix, the peptide, and for the complementary determining regions of the TCR in TCR-unbound states as compared to TCR-bound states. In this regard, Tedeschi and co-workers showed for the first time using a combination of a computer molecular dynamics simulation and in vitro experimentation that HLA-B*27:05, the strongest risk factor for the immune-mediated disorder ankylosing spondylitis (AS), was able to elicit anti-viral CD8+ T cell immune-responses even when the binding groove seemed to be only partially occupied by the Epstein Barr Virus epitope (pEBNA3A-RPPIFIRRL) [24]. In contrast, the non-AS-associated B*27:09 allele, distinguished from the B*27:05 by the single His116Asp polymorphism, was unable to display this peptide and therefore did not unleash specific CD8+ T cell responses in healthy subjects. The authors suggested that even partially filled grooves involved in peptide binding and presentation to CD8+ T cell receptors should be considered as part of the B27 immunopeptidome in evaluating viral immune-surveillance and autoimmunity. 

HLA-DQA1*05 and -DQB1*02 alleles encoding the DQ2.5 molecule and HLA-DQA1*03 and -DQB1*03 alleles encoding DQ8 molecules are strongly associated with celiac disease (CD) and type 1 diabetes (T1D). Farina and co-workers demonstrated previously that DQ2.5 genes showed a higher expression with respect to non-CD associated alleles in heterozygous DQ2.5 positive (HLA DR1/DR3) antigen presenting cells of CD patients. They showed that the HLA-DQA1*05 and -DQB1*02 alleles were co-ordinately regulated and expressed as a haplotype at significantly higher levels than non-predisposing alleles [25]. A different study of HLA DQ in T1D by Vadva and co-workers reports on a pedigree-based method for the haplotype analysis of the SNPs in and around the HLA-DR, DQ region using an optimised selection of SNP data to test whether SNPs inside and outside the gene regions are as useful for haplotyping as using HLA-typed alleles [26]. This new pedigree-based methodology for generating edited, non-ambiguous SNP haplotype phasing of minor allele frequency variation as found in the T1DGC pedigree resource might be useful in HLA SNP typing for association with various genetic phenotypes including autoimmune diseases such as T1D.

Experimental allergic encephalomyelitis (EAE) models are being developed in the rhesus monkey and cynomolgus macaque to elucidate the role of Epstein Barr Virus and MHC-E molecules in the presentation of encephalitogenic MOG peptides in multiple sclerosis [17]. The nonclassical HLA-E Class Ib molecules exhibit regulatory functions in both innate and adaptive immune responses and act as indicators for “missing-self” by continuously presenting peptides derived from signal sequences from HLA classical Class Ia molecules. HLA-E presents a 9-mer peptide derived from the signal sequences of HLA-A, -B, -C, and -G proteins to the CD94/NKG2 receptor that transduce an inhibitory signal to NK cells. In addition, it can bind and present antigenic peptides derived from bacterial and viral pathogens to HLA-E restricted CD8+ T cells that secrete antiviral cytokines and kill infected cells [17]. Rohm and co-workers reported in this Special Issue that, although limited, HLA-E polymorphism is associated with susceptibility to BK polyomavirus nephropathy (PyVAN) after a living-donor kidney transplant [27]. Their statistically significant findings suggest that a predisposition based on a defined HLA-E marker is associated with an increased susceptibility to developing PyVAN, and that assessing HLA-E polymorphisms may enable physicians to identify patients who are at an increased risk of this viral complication.

Yao and co-authors reported on the distribution of killer-cell immunoglobulin-like receptor genes and combinations of their HLA ligands in 11 ethnic populations in China [28]. The KIR and its HLA ligands exhibited diverse distribution and characteristics, where each group had its specific KIR and KIR–HLA pair profile. These findings could be expanded on in future population studies on the differential role of these receptors in health and disease.

Neuronal MHC-I has a role in synaptic plasticity, brain development, axonal regeneration, neuroinflammatory processes, and immune-mediated neurodegeneration. In the spinal cord, the MHC-I and beta-2 microglobulin (B2M) transcripts and proteins are upregulated after generating a peripheral motoneuronal lesion. In this Special Issue, Cartarozzi and co-workers presented their experimental findings that, after a ventral root crush, synaptic stripping and neuronal loss occurred more severely in B2M knockout (B2M-KO) mice than wild type mice [29]. Enhanced synapse detachment in B2M-KO mice was attributed to a preferential removal of inhibitory terminals, and the authors concluded that MHC-I molecules are important for a selective maintenance of inhibitory synaptic terminals after lesion formation, and that, with the absence of functional MHC-I expression in the B2M-KO mice, glial inflammatory reactions resulted in a more pronounced synaptic detachment in and around the lesion.

## 4. Breeding and Conservation: MHC Association with Reproductive Traits, Mate Choice and Fitness

Thirty-six years ago, Jones and Partridge suggested that the MHC is a system primarily for sexual selection and avoidance of inbreeding with histocompatibility fulfilling a secondary role [38]. However, to this day, the evidence for a role of the MHC as a life history gene complex with pleiotropic actions affecting reproduction and other fitness components such as mate selection, fecundity and survival remains relatively inconsistent and debatable. Some controversial aspects of the role of the MHC sexual selection and reproduction in primates [17], birds [18] and fish [19] are reviewed in this Special Issue. Three research papers specifically report on the MHC association with reproductive traits and kin selection (MHC-based mate choice) and fitness [30,31,32]. Ando and co-authors examined the association between Class II haplotypes and reproductive performances such as fertility index, gestation period, litter size, and number of stillbirths in the highly inbred population of Microminipigs [30]. They found statistically significant differences between haplotypes and the fertility index of dams, litter size at birth, litter size at weaning of dams, and body sizes of adult animals. Their findings suggest that MHC Class II genes of Microminipigs can affect some aspects of reproduction and therefore could be used as differential genetic markers for further haplotype and epistatic studies of reproductive traits and for improving selective breeding and fitness programmes.

Lan and co-workers described the use of MHC haplotypes as adaptive markers in their study of the relative roles of selection and genetic drift in seven populations of the endangered crested ibis [31]. They concluded that genetic drift had a predominant role in shaping the genetic variation and population structure of MHC haplotypes in bottlenecked populations, although some populations showed elevated differentiation of the MHC due to limited gene flow. The seven populations were significantly differentiated into three groups with some groups showing genetic monomorphism attributed to founder effects. The MHC haplotype results allowed the authors to propose various strategies for future conservation and management of the endangered crested ibis.

Zhu and co-workers used ten MHC loci as haplotypes and seven microsatellites outside the MHC region to test three hypotheses of female mate choice in a 17-year study of the giant panda [32]. They found female-choice for heterozygosity and disassortative mate choice at the inter-individual recognition level and that the MHC haplotypes were the mate choice target and not any of the seven microsatellite markers outside the MHC genomic region. They concluded from their long-term field, behavioural and genetic study that the MHC genes of giant pandas should be included when studying MHC-dependent reproductive studies. In this regard, the giant pandas [32] and the minimicropigs [30] appear to be two unique inbred mammalian models for investigating the correlation between the MHC and reproduction.

## 5. MHC Genomic Alleles (SNPs) and Haplotypes

An important subtheme to emerge from this Special Issue is that the association between MHC genomic SNP sequences and diseases, infections and phenotypes should be examined more often in the context of haplotypes (phased) rather than just genotypes (unphased). Two of the pioneers of human MHC haplotype research, Roger L. Dawkins who coined the term “Ancestral Haplotypes” and Chester Alper (and colleagues) who originated the term “Conserved Extended Haplotypes”, both published articles in this Special Issue showing that human population variation studied at the MHC haplotype level is a key requirement to better understanding the role that the MHC and its various genes and subregions may have in human traits including those of health and disease [16,26]. It is noteworthy that, apart from SNPs at gene loci, HLA interspersed indels such as the Alu, SVA, HERV and LTR retroelements also are useful MHC haplotype markers for differentiating between worldwide populations and for case-control stratification in disease association studies [39,40,41]. The benefits and disadvantages of assessing haplotypes as phased combinations of multilocus alleles instead of genotypes, single locus alleles or diplotypes were considered also in the reviews of MHC genetic diversity of primates, birds and fish [17,18,19]. In regard to the research articles, Farina and co-workers highlighted the importance of analysing the coordinated haplotypic expression of HLA-DQA and -DQB to better understand susceptibility to the autoimmune diseases T1D and CD [25]. Ando and colleagues used the MHC Class II haplotypes determined from breeding records of highly inbred Microminipigs to investigate their association with reproductive traits [30]. Lan and co-workers described the use of MHC haplotypes as adaptive markers in their study of the relative roles of selection and genetic drift in seven populations of the endangered crested ibis [31]. Zhu and co-workers used ten MHC loci as haplotypes and seven microsatellites outside the MHC region to test three hypotheses of female mate choice in a 17-year study of the giant panda [32]. Many of the reviews and research articles in this Special Issue demonstrate that there is a growing trend towards MHC haplotype analysis rather than simply limiting most genetic/phenotypic associations to only alleles or SNPs. 

## 6. Conclusions

The 18 papers gathered together in this Special Issue highlight the enormous genetic diversity and broad complexity of the MHC regulatory system and why its genomic structure and function is continuously under scientific investigation. These articles provide new insights as well as confirm some of the more tenuous and/or established beliefs about the genetic and biological roles of the MHC [16,17,18,19,20,21,22,23,24,25,26,27,28,29,30,31,32,33]. More importantly, many of these articles point MHC researchers and scholars in new directions where technical developments and research can greatly improve our knowledge and concepts of the structure and function of the MHC genomic region, especially as functional haplotypes in humans and all the other vertebrate species on the planet that thrive or are in danger of extinction. Some endangered species already need the assistance of researchers, breeders, and conservationists to use informative MHC genetic markers to help establish outbred colonies and families for their conservation and survival.
